# Stress and coping mechanisms of officers of the South African Police Service based in Tzaneen, Limpopo province, South Africa

**DOI:** 10.4102/sajpsychiatry.v25i0.1342

**Published:** 2019-11-27

**Authors:** Makhawukana R.V. Mushwana, Indiran Govender, Kathryn Nel

**Affiliations:** 1 Department of Psychology, University of Limpopo, Sovenga, South Africa; 2 Department of Family Medicine, Sefako Makgatho University, Medunsa, South Africa

**Keywords:** occupational stress, coping, organisational stressors, emotional disconnection, maladaptive coping strategies

## Abstract

**Background:**

In this study, stress is conceptualised as a psychological syndrome in response to stressors. Stress and inappropriate coping mechanisms constitute a serious problem in police profession. Organisational causes of stress, such as lack of support from management, are additional stressors. The ability to cope with this stress is influenced by marital status, gender, rank, age and years of service. In managing stress, police officers may use adaptive or maladaptive coping mechanisms.

**Aim:**

To investigate stress and the coping mechanisms used by police officers.

**Setting:**

The study was set in Tzaneen, Limpopo province, South Africa.

**Methods:**

This cross-sectional study used a self-administered questionnaire based on the Police Stress Inventory (PSI) and Coping Orientation to Problems Experienced (COPE) tools.

**Results:**

Of the 104 participants, female police officers had significantly higher stress intensity scores, while constables and captains had statistically significant lower stress scores. The highest ranked stressors were killing someone in the line of duty, a fellow officer killed in the line of duty, and knowledge of and experiencing the death of a fellow officer in the line of duty. The five most frequently occurring stressors were organisational in nature. Respondents were more likely to use coping strategies that were problem-focused.

**Conclusion:**

Organisational stressors were common, suggesting that the South African Police Services (SAPS) management should provide interventions that reduce the effects of work-related stressors. The majority of respondents used problem-focused coping strategies, indicating that most handle stressors in a positive manner.

## Introduction

Stress in this study is conceptualised as a psychological syndrome in response to stressors. Stress and inappropriate coping mechanisms constitute a serious problem in police profession.^1^ Police officers experience stress because of the nature of their work and the influences associated with the working environment.^1,2^

The components of stress and related coping mechanisms are changing in the South African police environment.^3^ The working environment of the South African Police Services (SAPS) is characterised by increasing violence, high crime rates and increasing murders of police officers.^3^ The work is more dangerous and stressful than in Europe or North America.^2,4^

Depression, post-traumatic stress disorder (PTSD) and suicidal behaviour are often found in police officers, particularly those who have spent many years in the force.^5^ Many police officers become traumatised as they witness community violence, motor vehicle accidents and shootings, some of which have taken the life of their peers.^3,6,7^ Stress among the SAPS officers has led to an increase in police brutality.^8^

The lack of support from management, perceived unfair promotion opportunities, poor working conditions, alleged unfair disciplinary processes, poor interpersonal relationships with colleagues and low salaries are among the organisational causes of stress in the SAPS.^7^ Levels of stress in the SAPS officers are also related to family pressures at home, inadequate counselling services and insufficient social and emotional support.^2,3,9^ In the light of this excessive stress, it is important for police officers to develop appropriate coping mechanisms.^2^

Police officers perceive the SAPS as being unable to support and protect its members.^10^ Gender, culture and race have a significant impact on their experience of stress.^1,11,12,13^ Police officers in South Africa feel that their contributions to the organisation are valueless, but they are not able to discuss this with their supervisors because they are of a different gender or ethnic group.^11^ Although female numbers in the SAPS have increased, women are still under-represented.^14^ South Africa has a patriarchal society; females at times have to deal with sexual harassment, public stereotypes and need to gain acceptance from male officers.^15^ The organisational culture inherent in policing is disadvantageous for female police officers.^16^ Nonetheless, female officers generally use more constructive coping mechanisms, and are more likely to rely on their faith and talking to their spouses in this regard. This is related to the perception in many cultures that females are expected to express their feelings, while males are not.^17,18^ Police officers are expected to encounter any situation with high levels of emotional control and a perceived lack of control is often the most difficult stressor for police officers.^17^ However, they often have little control over potential crisis, and thus find it difficult to deal with the situation.^19,20^

Police officers have a work environment that requires them to suppress their emotions related to shock, horror or sadness (emotional distance in order to properly deal with conflict and crime scenes).^3^ A display of emotions could make an officer alienated from his working colleagues as she/he may be seen as too ‘soft’.^21^ Officers are also expected to show compassion and understanding towards victims of crime; thus, they constantly have to switch between being empathetic, emotionless and, in some cases, being overly assertive, which could lead to cognitive confusion.^22,23^

Police officers in South Africa’s townships often cannot share their work issues (because of confidentiality) with peers, friends and family members.^24^ Within their home and family environment, police officers may be misunderstood, which places them under more stress and could result in feelings of isolation.^23^ One of the consequences related to the aforementioned issues is that there is a high divorce rate in the police force.^23^ All these factors lead to heightened stress levels.^23^ In extreme cases the feelings of isolation and despair can lead to suicide or possible famicide/suicide.

Some SAPS officers experience very high levels of stress and burnout, while others are less affected because they have the support of their families and possess inherent personality characteristics that reinforce their resilience.^25^ Officers who feel more supported are motivated in their work and more likely to show resilience.^25^ Differences in police ranks and stress intensity may exist but have not been well researched,^26^ although some research has shown that constables seem to suffer less stress than other ranks in the SAPS.^27^ Police officers with greater perceived work stress in the first year of police service have been found to show more severe depression.^28^

Antonovsky’s stress and coping model and Lazarus and Folkman’s interactional model of stress underpin this study.^29,30^ The interactive model considers other aspects related to personal resources (for instance, educational level and life experiences) that could influence an individual’s coping strategy.^20,31^

Police officers often use maladaptive coping strategies, such as taking non-prescription and prescription drugs, smoking, excessive alcohol intake and overeating.^32,33,34^ Such police officers have a greater chance of developing physical and psychological health problems.^34^ By contrast, police officers who use adaptive coping strategies, such as exercising and going for counselling, manage stress and anxiety in a better way.^23^

The main approaches towards coping among SAPS officers are either emotion-focused or problem-focused (using re-appraisal and minimal avoidance).^27^ In many African police forces, the use of withdrawal or avoidance (passive) coping strategies is associated with high levels of stress and burnout, while lower burnout levels are associated with constructive or active coping strategies.^27^ However, self-coping strategies have the best outcomes and interventions when linked to critical stress-debriefing, which is specifically tailored to the various types of incidents that police officers encounter.^35,36^ It has also been found that religiosity, as a personal coping resource, is utilised by many police officers who pray when handling traumatic cases.^36^ Religious coping styles are associated with positive mental health, while those with low religious beliefs are more inclined towards suicide.^37^

Police organisations in first world countries provide high-quality training and other resources to help police officers.^28^ However, even when such training is provided police officers still need counselling and sometimes psychological therapy after dealing with life-threatening and dangerous incidents.^38^ This prevents permanent mental and physical health problems.^38^ Provision of intervention programmes, which includes counselling and life-skills training, may assist both officers and their families to cope with work-related stress.

An investigation of suicidal ideation among SAPS officers using the Police Stress Inventory (PSI), Coping Orientation to Problems Experienced (COPE) tool, the Adult Suicide Ideation Questionnaire (ASIQ) and the Personality Characteristics Inventory (PCI) has concluded that low scores on approaches to coping, personality factors, such as conscientiousness and emotional instability, as well as high scores on avoidance coping were linked to suicidal ideation.^37^ Events with the highest correlation with stress intensity include violence in the line of duty and being involved in a motor vehicle accident (MVA) with an SAPS vehicle.^2^ Police officers who show resilience are those selected through effective pre-employment selection strategies and those who undergo intensive repetitive training techniques.^39^

It is apparent that internationally and in South Africa, police officers are vulnerable to many stressors.

Therefore, we sought to investigate stress and coping mechanisms of police officers in the remote region of Tzaneen, Limpopo province. The Greater Tzaneen Local Municipality is a Category B municipality situated in the eastern quadrant of the Limpopo province within the Mopani District. It contains 125 rural villages, with almost 80% of households residing in these villages. It has both urban and rural settings. Its main economic driver is agriculture followed by tourism. The Greater Tzaneen Municipality has a population of 390,095. The majority of population is aged between 20 and 24 years, and there are more females (53%) than males. Unemployment rate ranges from 36.9% to 47.1%. The Greater Tzaneen Municipal area has the highest crime rate for Limpopo.^5^

## Research methodology and design

### Setting

The design employed a cross-sectional survey using self-administered questionnaires. The study population comprised police officers stationed at police stations in the Greater Tzaneen Municipality (Maake, Ritavi, Letsitele and Tzaneen police stations). Stratified sampling was used to ensure adequate representation of all police ranks. The total population was 317 and a representative sample size was calculated to be 175 using Krejcie and Morgan’s model.^40^

Each officer, randomly selected from each sub-group, was sent an invitation letter, after which questionnaires were sent to those who consented to participate. Data were collected from 05 December 2015 to 22 January 2016.

### Study instruments

The COPE and PSI, which are reliable and valid standardised instruments, were used. The survey questionnaire had three parts: demographic section; PSI and the COPE questionnaire. The PSI, developed by Pienaar and Rothman,^41^ has been used frequently and is found to be valid. The three internally reliable factors included (1) stress from job demands (*α* = 0.92); (2) stress from lack of job resources (*α* = 0.92) and (3) police-specific stressors (*α* = 0.89).^42^ Each item describes a job-related stressor event and assesses both the perceived severity and the frequency of occurrence of that event.

The COPE inventory is a multi-dimensional 75-item questionnaire that assesses the different ways in which individuals cope under different circumstances.^43^ COPE is scored on a four-point rating scale.^43^ The COPE inventory has a Cronbach’s α of 0.88. Concurrent validity analyses indicated that these factors assess self-efficacy for different types of coping.^43^

### Data analysis

Descriptive statistics were used to analyse the univariate data. The Pearson (*p*) correlation coefficient was used to analyse relationships between variables. An independent t-test was used to test for any significant stress and coping differences between the demographic groups. The significance level was set at 0.05.

### Ethical considerations

Permission for the study was obtained from the Director SAPS Limpopo province. The University of Limpopo Ethics Committee provided ethical clearance vide reference number: TREC/32/2014:PG. Anonymity and confidentiality were maintained throughout the research process and informed consent was obtained from all the participants, who were debriefed.

## Results

The response rate was 59% (104 participants). All demographic sub-groups were represented. There were *N* = 26 (25%) females and most respondents were constables (*N* = 47; 45.2%) (see Table 1). Female police officers were generally under-represented and more so from higher ranks (refer to Table 2).

**TABLE 1:** Demographic characteristics of respondents (*N* = 104).

Demographic characteristics		Number	Percentage
Race	Indian	9	8.7
	Mixed race	5	4.8
	White	6	5.8
	Black	84	87.4
Religion	Christian (African)	67	64.4
	Muslim (Indian and mixed race)	14	13.5
	Not reported	23	22.1
Marital status	Married	58	55.8
	Single	46	44.2
Rank	Constable	47	45.2
	Sergeant	18	17.3
	Inspector	28	26.9
	Captain	8	7.7
	Colonel	3	2.9
Age in years	18–35	46	44.2
	36–65	58	55.8

**TABLE 2:** Gender and rank of respondents.

Rank	Number of male officers	Percentage	Number of female officers	Percentage	Total (%)
Constable	34	32.7	13	12.5	45.2
Sergeant	15	14.4	3	2.9	17.3
Inspector	25	24.0	3	2.9	26.9
Captain	5	4.8	3	2.9	7.7
Colonel	2	4.8	1	0.9	2.9

**Total**	**81**	**77.9**	**23**	**22.1**	**100.0**

### Police stress inventory (PSI)

Female police officers had significantly higher stress intensity scores (*p* < 0.01) compared to males.

Those police officers who expressed adherence to Christian faith were constables and captains and statistically had significantly lower stress scores (refer to Table 3).

**TABLE 3:** Demographic data and stress intensity scores.

Demographics	Stress intensity	s.d.	Number	*p*
**Gender**				0.01
Female	64.53	12.29	26	-
Male	55.48	15.01	78	-
**Rank**				0.01
Constable	53.17	15.65	47	-
Sergeant	64.96	11.99	18	-
Inspector	61.2	12.34	28	-
Captain	54.51	8.41	8	-
Colonel	62.36	16.00	3	-
**Religion**				0.04
Christian	55.85	14.88	67	-
Muslim	61.35	12.22	14	-
No affiliation	60.72	16.12	23	-
**Age**				0.59
18–35 years	56.3	14.27	46	-
36–65 years	59.4	16.25	58	-
**Marital status**				0.57
Married†	59.4	16.25	58	-
Single‡	56.3	14.27	46	-
**Language**				0.04
Xitsonga	36.46	15.16	48	-
Sepedi	57.88	13.53	36	-
English	63.01	14.66	20	-

s.d., standard deviation.

† , Living together and married respondents’ scores were combined into the married group.

‡ , Divorced, single and widowed respondents’ scores were combined into the single group. This allowed for a more comprehensive analysis of demographic variables. Participants in this category did not have a partner at home. Killing someone in the line of duty and a fellow officer killed in the line of duty were the highest ranked stressors (refer to Table 4).

The five most frequently occurring stressors over the 6 months preceding the onset of the study were all organisational in nature as listed in Table 5.

**TABLE 4:** Police stressors in rank order (organisational and non-organisational).

Rank order	Police stressors	*M*	s.d.
1	Killing someone in the line of duty (non-organisational)	4.66	0.79
2	A fellow officer killed in the line of duty (non-organisational)	4.55	0.86
3	Inadequate support by supervisor (organisational)	3.79	1.17
4	Fellow workers not doing their job (organisational)	3.70	1.14
5	Excessive paperwork (organisational)	3.42	0.95
6	Having to handle a large crowd/mass protest	3.38	1.07
7	Making critical on-the-spot decisions (non-organisational)	3.31	1.15
8	Insufficient personnel to handle an assignment (organisational)	3.30	1.27
9	Lack of participation in policymaking decisions (organisational)	3.28	1.15
10	A forced arrest or being physically attacked (non-organisational)	3.27	1.11
11	Having to go to court (organisational)	3.23	1.10
12	Staff shortage (organisational)	3.20	1.24
13	Inadequate or poor-quality equipment (organisational)	3.17	1.18
14	Lack of recognition for good work (organisational)	3.13	1.22
15	Period of inactivity (organisational)	3.11	1.08
16	Having to deal with the media (organisational)	3.04	1.46
17	Conflicts with other departments (organisational)	3.03	0.99
18	Personal insults from customer/colleague(s) (organisational)	3.00	1.25
19	Poorly motivated co-workers (organisational)	2.97	1.08
20	Difficulty in getting along with supervisor(s) (organisational)	2.97	1.04
21	Frequent interruptions (organisational)	2.96	1.38
22	Assignment of new or unfamiliar duties (organisational)	2.93	1.31
23	Assignment of increased responsibility (organisational)	2.92	1.18
24	Lack of opportunity for advancement (organisational)	2.92	0.98
25	Poor or inadequate supervision (organisational)	2.92	1.07
26	Racial conflict (non-organisational)	2.92	1.16
27	Reorganisation and transformation within the organisation (organisational)	2.87	1.21
28	Shift work (organisational)	2.86	1.25
29	Attending to incidences of domestic violence (non-organisational)	2.82	1.22
30	Too much supervision (organisational)	2.77	1.04
31	Working overtime (organisational)	2.77	1.15
32	Performing tasks not in my job description (organisational)	2.73	1.13
33	Experiencing negative attitude towards the organisation (organisational)	2.72	1.01
34	Covering for another employee (organisational)	2.72	1.15
35	Meeting deadlines (organisational)	2.70	0.98
36	Delivering bad news (non-organisational)	2.70	1.10
37	Competition for advancement (organisational)	2.69	1.15
38	Insufficient personal time (for coffee, lunch) (organisational)	2.65	1.10
39	Frequent changes from boring to demanding activities (organisational)	2.69	1.25
40	Noisy work areas (organisational)	2.65	1.10
41	Assignment of disagreeable duties (organisational)	2.56	1.15
42	Dealing with crisis (non-organisational)	2.56	1.10
43	Seeing criminals go free (lack of evidence) (non-organisational)	2.56	0.98
44	Difficulty in getting along with colleagues (organisational)	2.55	1.15

s.d., standard deviation; *M*, mean.

**TABLE 5:** The most frequently occurring stressors in previous 6 months.

Stressor	*M*	s.d.
Fellow officers not doing their work	8.04	13.33
Inadequate or poor-quality equipment	5.70	3.37
Excessive paperwork	7.19	8.18
Poor or inadequate supervision	5.82	6.93
Staff shortages	7.21	9.81

s.d., standard deviation; *M*, mean.

### Coping inventory (COPE)

Coping strategies across different demographic variables are presented in Tables 6 and 7. Constables and sergeants preferred to use emotionally focused coping strategies.

**TABLE 6:** Demographic characteristics of officers using emotionally focused and problem-focused coping strategies.

Characteristics	s.d.		*M*		Frequency		*p*	
	EF	PF	EF	PF	EF	PF	EF	PF
**Sex**							0.275	0.300
Female	0.55	0.63	2.61	2.56	26	26	-	-
Male	0.60	0.57	2.55	2.44	78	78	-	-
**Rank**							0.065	0.69
Constable†	0.63	0.74	2.56	2.83	47	47	-	-
Sergeant†	0.36	0.89	2.93	3.19	18	18	-	-
Inspector	0.57	0.43	2.44	2.65	28	28	-	-
Captain	0.41	0.53	2.20	2.42	8	8	-	-
Colonel	0.71	0.29	2.62	3.04	3	3	-	-
**Religion**							0.825	0.0407
Christian	0.65	0.78	2.56	2.88	67	67	-	-
Muslim	0.43	0.59	2.58	2.74	14	14	-	-
No affiliation	0.46	0.65	2.53	2.82	23	23	-	-
**Age**							0.052	2.00
18–35 years	0.58	0.72	2.69	2.96	46	46	-	-
36–65 years	0.53	0.68	2.44	2.77	58	58	-	-
**Marital status**							0.52	2.00
Married	0.53	0.68	2.44	2.77	58	58	-	-
Single	0.58	0.72	2.69	2.96	46	46	-	-

s.d., standard deviation; *M*, mean; EF, emotionally focused; PF, problem-focused.

† , Constables and sergeants had a statistically significant difference (*p* = 0.003) in coping compared to other ranks.

**TABLE 7:** The most frequently used coping strategies.

Coping strategy	*M*	s.d.
I try to see it in a different light to make it seem more positive	2.96	0.98
I look for something good in what has happened	2.93	0.92
I try to come up with a strategy about what to do	2.92	1.00
I try to find comfort in my religion	2.88	1.02
I think hard about what steps to take	2.84	0.95
I think about how I might best handle the problem	2.83	1.01
I pray every day	2.82	0.98
I concentrate my efforts on doing something about it	2.76	1.07
I believe the ancestors will help me	2.75	0.97
I try to grow as a person as a result of experience	2.74	1.07

s.d., standard deviation; *M*, mean.

Table 7 indicates that regardless of their age, religion, language, gender, marital status or rank, all respondents used similar problem-focused coping strategies.

The 10 most commonly used coping strategies are listed in Table 8. Most of these were problem-focused, e.g. ‘I try to come up with a strategy about what to do; I think hard about what steps to take; and I think about how I might best handle the problem’.

Respondents were more likely to use problem-focused coping strategies (mean value 2.63; standard deviation [s.d.] 0.71) than emotionally focused coping strategies (mean value 2.51; s.d. 0.63) and avoidance coping strategies (mean value 1.88; s.d. 0.49) when addressing stress.

## Discussion

### Key findings

Women officers are under-represented in Tzaneen, despite the fact that it would be advisable that the demographics of the SAPS would mirror the general population in order to build trust.^14^ Women police officers experienced more job-related stress than male police officers, and identify stressors in a different manner from male officers.^17,18,44,45^ This research supports previous studies, as there were statistically significantly higher stress-intensity levels among female participants. The intensity of stress may be more severe for female officers because of issues such as sexual harassment, as reported in other research.^16^ We found that males and females used the same coping strategies (problem and emotional), which was similar to a US study.^45^

However, it has been reported elsewhere that generally males and females cope with stress differently: males use problem-focused strategies, while women use emotionally focused strategies.^46^

### Discussion

Appraisal of a stressor is a function of personality, beliefs, values, attitudes, support structures, goals and individual experiences.^47^ This, in turn, facilitates specific coping mechanisms in an individual. In this study, the following two statements were in the top 10 coping mechanisms: ‘I pray every day and I try to find comfort in my religion’. This suggests that many use religion as a coping mechanism. The coping mechanism response, ‘I believe the ancestors will help me’, is appropriate to the socio-cultural context of the respondents and might be linked to religious coping mechanisms with an emotional focus.

The expression, ‘I accept the reality of the fact that it happened’, implies that many respondents recognise the reality of a situation, and with that acceptance is likely to mobilise personal coping resources. This response is linked to: ‘I think about how I might best handle the problem’, the sixth most common coping strategy, suggesting that many respondents used problem-focused coping strategies. This strategy reinforces Lazarus’s model which highlights the role of cognitive appraisal in the perception of potential stressors when forming an appropriate coping response.^30^ Furthermore, it supports the study of occupational stress and work engagement of SAPS members which found that significant differences in individual responses to stress could be attributed to the process of cognitive appraisal.^2^ Coping strategies in the top 10 indicate the use of problem-focused strategies with a cognitive and behavioural component. For instance, ‘I try to come up with a strategy about what to do and I think hard about what steps to take’.

It is important to assess individual and situational factors as they may mediate the stressor.^30^ In this study, situational stress factors ranked among the top 10, and included: ‘Having to handle a large crowd/mass demonstrations’; ‘A forced arrest or being physically attacked’, and ‘Having to go to court’. Each of these situations is unpredictable. Indeed, the inherent nature of police work is unpredictable, ambiguous and uncertain. These situational factors linked to poor coping skills may result in stress.^2^

No significant differences in stress were found between different age groups. This is different from other studies where younger police officers experienced more stress.^48^ However, it is also reported that emotional exhaustion (burnout) of police officers exists across all age groups.^49^ Constables experienced less stress than other ranks in our study, which is underpinned by other research related to stress in SAPS officers. The reason for this finding might be linked to the fact that constables have worked in the police force for a shorter period than other officers, and thus have experienced less exposure to stressors.^27^ Another finding is that police officers also become frustrated and stressed when they experience difficulties in rank promotion.^24^

Occupational stressors stem from the problems implicit in police work.^50^ Five of the top 10 stressors in our study were organisational in nature, namely (1) inadequate support by supervisor; (2) fellow workers not doing their job; (3) excessive paperwork; (4) insufficient personnel to handle an assignment and (5) lack of participation in policymaking decisions. These stressors were generally managed with adaptive coping mechanisms using problem and emotionally focused strategies, as indicated by the following: ‘I try to see it in different light, to make it seem more positive’ and ‘I look for something good in what has happened’. These findings support those other researchers in South Africa who found that police officers experienced more psychological distress in relation to organisational stressors, for instance administrative issues and paperwork, as compared to operational problems.^2,23^

Career development for SAPS officers in the police force in the Greater Tzaneen Municipality does not appear to meet the respondents’ needs. This is reflected by the stressors ‘Lack of recognition for good work’ and ‘Lack of opportunity for advancement’. These organisational career constraints are well-known sources of stress and frustration in the police force.^23^

In general, police officers of Tzaneen do not use maladaptive and avoidance coping strategies, such as drinking alcohol or taking drugs, engaging in self-blame, denying that something has happened, and giving up when faced with stressful events. They use problem-focused strategies. These officers experienced maximum stress (from stressors such as ‘killing someone in the line of duty’, ‘a fellow officer killed in the line of duty’, and ‘inadequate support by supervisor’) when there was an imbalance between these stressors and their ability to cope. The stressor scores indicate that police officers perceive their occupational stressors within their environment as stressful and demanding. Previous research on SAPS officers reports that certain interventions, such as narrative writing and/or topic-specific training, led to reduction in physiological and psychological stress effects.^21^

### Strengths and limitations

The study was conducted with a small sample; however, the sample was representative of SAPS officers in the Greater Tzaneen Municipal area. The small sample size limits generalisations of these findings, especially when breaking the groups further into different genders or ranks. Burnout was not explored; in retrospect it would have been beneficial to explore burnout as an additional stress-related facet. Another limitation is that we did not use regression analysis to test relationships between variables because of our sample size.

Four police stations from Tzaneen region of Limpopo province were chosen to participate in the study, allowing for a diverse sample of respondents (ethnicity and gender). All the questionnaires used in this study were validated and standardised questionnaires which were used previously in South African surveys and had high measurements of internal consistency (Cronbach’s α).^42,43^ This increased the validity of results.

### Implications and recommendations

Organisational stressors seem to be very common and this suggests that the SAPS management should provide interventions that reduce the effects of work-related stressors. The majority of these police officers use problem-focused coping strategies together with emotionally focused coping strategies. This result indicates that most respondents handle stressors in a positive manner.

Further research needs to be conducted on the selection process of police officers, with a focus on personality, coping styles and resilience.
